# Molecular basis of precision nutrition: Food components, microbiome-derived metabolites, and multi-omics modeling

**DOI:** 10.1016/j.fochms.2026.100411

**Published:** 2026-05-08

**Authors:** Souvia Rahimah, Trina Ekawati Tallei, Maghfirah Savitri, Chika Yamada, Hyo Jung Kim, Min Choi, Moon Nyeo Park, Youdiil Ophinni, Bonglee Kim

**Affiliations:** a Department of Food Industrial Technology, Faculty of Agroindustrial Technology, Universitas Padjadjaran, Jatinangor, West Java, Indonesia; b Department of Biology, Faculty of Mathematics and Natural Sciences, Sam Ratulangi University, Manado 95115, North Sulawesi, Indonesia; c Robert Wolter Monginsidi Army Hospital, Manado, North Sulawesi, Indonesia; d Department of Environmental Coexistence, Center for Southeast Asian Studies, Kyoto, Japan; e Department of Pathology, College of Korean Medicine, Kyung Hee University, Seoul 02447, Republic of Korea

**Keywords:** Precision nutrition, Metabolic health, Multi-omics, Gut microbiome, Machine learning

## Abstract

Metabolic disorders, including obesity, type 2 diabetes, metabolic syndrome, and fatty liver disease, reflect multifactorial interactions among diet, host genetics, the environment, and the gut microbiome. However, conventional population-level dietary guidance often fails to capture the marked interindividual variability in metabolic responses to identical foods and nutrients. Precision nutrition has therefore emerged as an integrative paradigm that combines genomics, epigenomics regulation, microRNA-mediated control, and microbiome profiling to refine dietary recommendations, with a growing emphasis on targeted functional food-based strategies for metabolic health. This narrative review synthesizes the mechanistic foundations of precision nutrition, emphasizing how functional foods and their bioactive constituents engage nutrient-sensing and regulatory pathways that connect dietary exposures to gene regulation and downstream metabolic phenotypes. We summarize evidence for diet–gene interactions at key metabolic loci, epigenetic programming shaped by early-life nutrition, and diet-responsive microRNAs as candidate biomarkers of nutritional response. We further examine microbiome-derived signaling, specifically short-chain fatty acids and bile-acid metabolism within the gut–liver axis, as a major route by which functional dietary components can influence host pathways and condition metabolic outcomes. We highlight insights from large cohorts and controlled metabolic profiling studies and discuss enabling methodological advances, including multi-omics integration, causal inference, and machine learning models for response prediction. Key limitations remain, notably incomplete reproducibility, heterogeneity in exposure and outcome measurements, data governance challenges, and barriers to clinical implementation. In summary, precision nutrition provides a biologically grounded framework for personalized functional-food and dietary strategies, but robust validation and responsible translation are required before routine adoption.

## Highlights

• Multi-omics data link diet, microbiome, and host molecular variability.
• AI/ML models predict diet response and identify responder subgroups.
• Microbiome metabolites (SCFAs, bile acids) signal to metabolic pathways.
• Personalized diets aim to improve glycemia, lipids, and inflammation.

## Graphical abstract

Population-level dietary guidance often yields variable metabolic responses because individuals differ in host molecular regulation and diet–microbiome interactions. Precision nutrition integrates food and dietary exposures with host genomics, including single nucleotide polymorphisms (SNPs), epigenetic regulation, microRNA-mediated control, gut microbiome features, and metabolic biomarkers captured by multi-omics profiling. These heterogeneous data streams, together with lifestyle and environmental factors, are analyzed using bioinformatics and AI-enabled models to derive response signatures and predict individual response to foods and nutrients. The resulting outputs inform personalized dietary strategies, including optimized food selection, nutrient distribution and timing, and functional-food or bioactive-rich dietary patterns, supported by digital monitoring via smartphone apps and wearable sensors to promote sustained lifestyle change. This data-to-decision workflow provides a mechanistically grounded route for stratified dietary guidance to improve glycemic regulation, lipid handling, and inflammatory tone.

**Figure:**
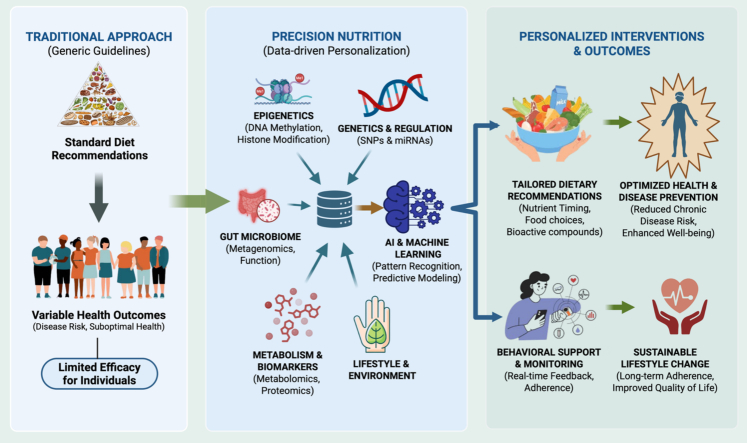

## 1 Introduction

Metabolic disorders, including obesity, type 2 diabetes, metabolic syndrome, and non-alcoholic fatty liver disease (NAFLD), which is now increasingly conceptualized as metabolic-dysfunction associated fatty liver disease (MAFLD) or metabolic dysfunction-associated steatotic liver disease (MASLD), have emerged as dominant contributors to global morbidity and mortality. Metabolic syndrome affects nearly one-third of the world's population and confers a substantially elevated risk of cardiovascular disease and premature death (Engin, 2017). In parallel, the prevalence of fatty liver disease has increased alongside rising rates of obesity and diabetes, with recent meta-analyses estimating a global prevalence of 30–38% and forecasting continued growth in the absence of effective intervention (Teng et al., 2022). Importantly, MASLD is no longer viewed as a liver-restricted condition but as a hepatic manifestation of systemic metabolic dysfunction, closely linked to insulin resistance, dyslipidemia, and cardiovascular events (Lekakis & Papatheodoridis, 2024). These intersecting pathways underscore the need for integrative preventive and therapeutic strategies that account for nutritional, genetic, and environmental determinants of metabolic health.

Functional foods have gained increasing attention as a dietary approach for supporting metabolic health. Regulatory and public health bodies, including the European Food Safety Authority (EFSA), the Food and Drug Administration (FDA), and the World Health Organization (WHO), define functional foods as conventional foods or food components that, beyond fulfilling basic nutritional requirements, deliver bioactive constituents capable of conferring physiological benefits or lowering the risk of chronic conditions such as diabetes and cardiovascular disease (Fekete et al., 2025). This concept is distinct from nutraceuticals, which generally refer to isolated or concentrated bioactive substances formulated as supplements. In contrast, functional foods, whether in their native state or deliberately formulated and enriched with specific bioactive ingredients, are consumed as part of the habitual diet (Faienza et al., 2024). In the context of metabolic health, functional foods offer practical advantages, including greater dietary adherence and the opportunity for additive or synergistic interactions among bioactive compounds within their native food matrix, which may enhance biological efficacy (Zhao et al., 2025).

Although a substantial body of research has investigated the effects of functional foods, such as polyphenols, probiotics, unsaturated fatty acids, and functional fibers, on key metabolic outcomes like insulin sensitivity, lipid metabolism, and gut microbiota composition (Bock et al., 2021; Jia et al., 2024; Meiners et al., 2025), individual responses to these dietary interventions remain highly variable. This heterogeneity highlights the importance of genetic and epigenetic determinants of nutritional responsiveness. Variants, such as single nucleotide polymorphisms (SNPs) in genes involved in metabolic regulation, can modify individual sensitivity to dietary bioactive compounds. In parallel, epigenetic processes, including DNA methylation, histone modifications, and regulation by non-coding RNAs such as microRNAs (miRNAs), provide mechanistic links between dietary exposure and sustained changes in gene expression. These emerging dimensions of nutritional science, commonly described as nutriepigenomics and nutrimiromics, have become integral to contemporary nutrigenomic research (Bacaloni & Agrawal, 2025; Quintanilha et al., 2017; Siddeek & Simeoni, 2022). Furthermore, diet-microbiome interactions generate signaling metabolites, including short-chain fatty acids and microbially modified bile acids, that engage host nutrient-sensing pathways to further shape metabolic phenotypes.

Despite the growing interest, the integration of functional food interventions with these multi-omics layers in metabolic disease remains limited, particularly in well-designed, long-term human studies (Ostaiza-Cardenas et al., 2025). Most studies lack stratification based on genetic variation or epigenetic profiles, and few have examined changes in miRNA expression as potential mediators of dietary effects. This methodological gap constrains the understanding of the biological basis underlying interindividual variability in responses to functional foods and limits insight into the molecular pathways through which such variability arises (Fekete et al., 2025). Integrating evidence from nutrition science with genomic, epigenetic, miRNA, and microbiome research is therefore essential for developing more mechanistically informed and personalized approaches in future nutritional studies (Ramos-Lopez et al., 2022; V. Singh, 2023).

This narrative review synthesizes recent advances in functional food-based strategies to support metabolic health, emphasizing interindividual molecular determinants of dietary response, including genetic variation, such as SNPs, epigenetic regulation, and miRNA-mediated control. We critically appraise the current evidence, highlight methodological and translational gaps, and propose priorities for future research to enable robust, multi-omics-informed precision nutrition frameworks for risk reduction and metabolic disease management.

## 2 Conceptual framework of precision nutrition

Precision nutrition is grounded in the concept that metabolic phenotypes arise from continuous interactions among genetic background, dietary intake, environmental exposures, and the gut microbiota (Kirk et al., 2021; Mansour et al., 2024). Within this framework, individual genetic variation, including SNPs in genes governing metabolic regulation, inflammatory signaling, and xenobiotic metabolism, influences how dietary components are sensed, processed, and translated into biological responses (Mansour et al., 2024; Phillips, 2013). Environmental factors, such as lifestyle patterns, physical activity, and exposure to pollutants, further modulate metabolic homeostasis and interact with both genetic makeup and dietary inputs (Khalil et al., 2023; Tiffon, 2018). The gut microbiota adds a regulatory dimension by generating bioactive metabolites. For instance, microbes ferment dietary fibers into short-chain fatty acids (SCFAs) and bioconvert polyphenols into active phenolic metabolites. They also modify endogenous host molecules, such as converting primary bile acids into secondary bile acids (Man et al., 2020; Zhang et al., 2023). Such interconnected processes explain why identical dietary interventions can lead to substantially different metabolic outcomes across individuals, reflecting differences in genomic context and microbial composition (Berding et al., 2021; Fenech et al., 2011; Hussein et al., 2025).

To address this complexity, precision nutrition increasingly adopts integrative multi-omics approaches that combine genomics, transcriptomics, proteomics, metabolomics, epigenomics, and microbiome profiling through metagenomic analyses (Berciano et al., 2022; Livingstone et al., 2022; V. K. Singh et al., 2024). Genomic strategies, including SNP genotyping and whole-genome sequencing, inform inherited susceptibility and gene-nutrient interactions that influence dietary responsiveness (Ferguson et al., 2016; Mullins et al., 2020). Transcriptomic and proteomic analyses capture diet-related alterations in gene expression and protein abundance, providing insight into downstream molecular responses (Mahato et al., 2024). Metabolomics delivers a functional representation of metabolic status by profiling host- and microbiota-derived metabolites that reflect dietary exposure and physiological impact (Kortesniemi et al., 2023). Complementing these layers, epigenomic analyses, including DNA methylation and histone modifications, reveal how nutritional and environmental factors can induce sustained alterations in gene regulation (Abraham et al., 2023; Alegría-Torres et al., 2011). Integrating these datasets enables the examination of dietary effects across interconnected molecular networks rather than isolated associations. In this context, metabolomics has emerged as a particularly powerful tool for personalized nutrition, as it directly links nutrient intake to metabolic phenotypes with high analytical resolution (Kim et al., 2025).

Based on these principles, a modular model proposes that nutrient-sensing pathways such as AMPK, mTOR, PPARs, the insulin-IGF axis, and xenobiotic nuclear receptors operate as integrative hubs that converge signals from dietary components, microbial metabolites, and endogenous regulatory molecules (Jardon et al., 2022). Crucially, the function of these pathways as ‘integrative hubs’ explains both the convergence and divergence of metabolic responses observed in precision nutrition. On one hand, distinct environmental or dietary inputs, such as specific polyphenols (e.g., resveratrol, quercetin, or epigallocatechin gallate [EGCG]), microbiota-derived SCFAs, or physical activity, can converge on the same hub (e.g., activation of AMPK or PPARs), ultimately resulting in the same favorable metabolic phenotype, such as enhanced insulin sensitivity or improved lipid oxidation. Conversly, because individual genetic (such as SNPs in *PPARG* or *FTO*) and epigenetic variations (such as DNA methylation patterns at metabolic gene promoters) modify the activation threshold, sensitivity, and feedback control of these very same hubs, identical dietary inputs can yield highly heterogeneous physiological responses that are increasingly recognized as central to personalized nutrition. Within this framework, bioactive compounds, including these specific classes of polyphenols, influence multiple downstream signaling pathways such as NF-κB, AMPK, PI3K/AKT, and PPARs, thereby shaping metabolic regulation and inflammatory processes (Cano et al., 2024). Hence, polyphenols may reduce oxidative stress, enhance glucose uptake, promote fatty acid oxidation, and modulate lipid metabolism through pathways such as AMPK and NF-κB, while also affecting adipogenesis, cytokine production, gut microbiota composition, and incretin signaling (Bayram et al., 2018; Cano et al., 2024).

**Fig. 1:**
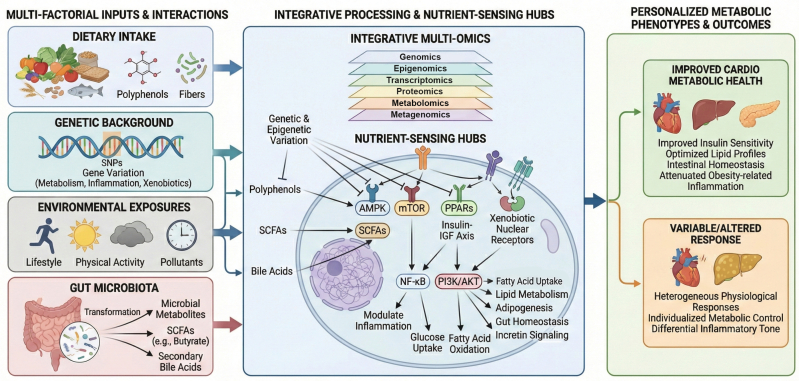
The modular model of precision nutrition illustrating how individual genetic, environment, and microbial factors integrate with dietary signal through multi-omics network and nutrient-sensing hubs to produce diverse metabolic phenotypes.

These combined actions support improvements in insulin sensitivity, lipid profiles, and intestinal homeostasis, contributing to cardiometabolic health. However, the capacity of a given polyphenol to activate AMPK signaling varies substantially across individuals due to differences in upstream regulatory pathways and host-specific genetic and epigenetic factors (Leziak et al., 2025; Morand, 2024; Price et al., 2012). In parallel, microbial metabolites, including SCFAs (such as butyrate) and modified bile acids, interact with host receptors to influence energy balance and lipid metabolism (Sankarganesh et al., 2025). Through these layered interactions, the modular model links nutrient and microbiome inputs to personalized outcomes in metabolic control and inflammatory tone (Zhou & Pang, 2025). As shown in Fig. 1, the modular model of precision nutrition illustrates how individual genetic, environmental, and microbial factors integrate with dietary signals through multi-omics networks and nutrient-sensing hubs to generate diverse metabolic phenotypes. Building on this framework, prior studies emphasize that a mechanistic investigation of interindividual variability is essential for guiding precision nutrition strategies that target multiple regulatory nodes rather than relying on uniform dietary prescriptions (Clemente-Suárez et al., 2025; Jamrat et al., 2023).

## 3 Genetic and epigenetic contributors to Interindividual variability in dietary responses

**Fig. 2:**
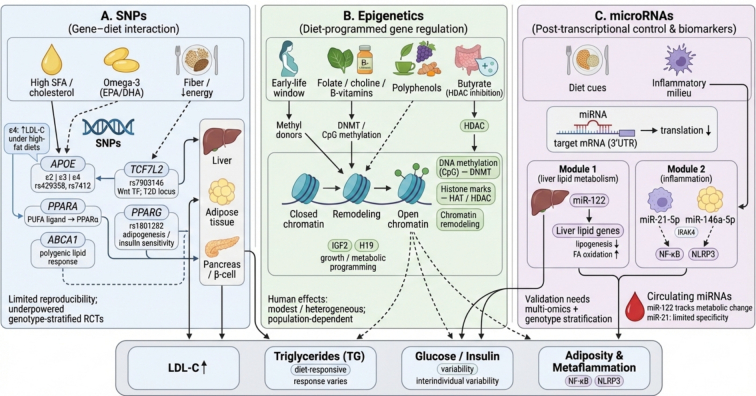
Multi-layer genetic regulation of dietary response. (A) SNPs in *APOE* (ε2/ε3/ε4; rs429358/rs7412), *PPARA*, *ABCA1*, *TCF7L2* (rs7903146), and *PPARG* (rs1801282) interact with diet (saturated fat/cholesterol, omega-3 PUFA, fiber/energy) to shape tissue metabolism (liver, adipose, β-cells) and variability in LDL-C, triglycerides, and glucose/insulin. (B) Nutrition modulates gene expression through DNA methylation (DNMT), histone modifications (HAT/HDAC), and chromatin remodeling, with heightened sensitivity in early life; methyl donors, polyphenols, and butyrate (HDAC inhibition) influence growth/metabolic programs (e.g., *IGF2*, *H19*). (C) Diet- and inflammation-responsive miRNAs regulate target mRNAs via 3′UTR binding; miR-122 affects hepatic lipid genes, while miR-21-5p and miR-146a-5p converge on inflammatory pathways (NF-κB, NLRP3). Across all panels, solid arrows (→) indicate direct activation, molecular interaction, or metabolic flow, flat-ended lines (⊣) denote pathway inhibition or suppression, and dashed lines represent indirect or multi-step mechanisms.

This section synthesizes evidence from genetic and epigenetic research to explain why individuals exhibit heterogeneous metabolic responses to dietary exposures. It focuses on three interrelated layers of regulation: inherited genetic variation (SNPs), diet-responsive epigenetic modifications (such as DNA methylation), and miRNA-mediated post-transcriptional regulation (including both endogenous and food-source miRNAs). These mechanisms illustrate how a fixed genetic background interacts with nutritionally sensitive regulatory processes to influence lipid and glucose metabolism, bioavailability of food compounds, and disease susceptibility**.** Fig. 2 presents a schematic overview of these regulatory layers and their interactions with dietary factors.

### 3.1 Single nucleotide polymorphisms (SNPs)

Single nucleotide polymorphisms in genes involved in lipid metabolism have long been recognized as key contributors to interindividual variability in lipid profiles and dietary lipid responsiveness. Among these, the *APOE* gene, which encodes apolipoprotein E (a major cholesterol carrier essential for lipid transport and clearance), is the most extensively studied. Its three common isoforms, ε2, ε3, and ε4, arise from specific combinations of two SNPs, rs429358 and rs7412, which dictate the functional differences observed across clinical populations (Najd-Hassan-Bonab et al., 2023). Carriers of the ε4 allele consistently exhibit higher LDL cholesterol levels and less favorable lipoprotein profiles, particularly under high-fat dietary conditions, compared with ε3 carriers (Ioannidou et al., 2024). Beyond circulating lipid levels, the *APOE* genotype has been shown to influence hepatic lipidomic patterns in hepatocytes, indicating variant-specific differences in intracellular lipid handling and mitochondrial function (Almeida et al., 2024). The ε4 allele has also been linked to heightened sensitivity to dietary cholesterol and saturated fat intake, contributing to greater variability in plasma lipid responses and cardiometabolic risk (Clemente-Suárez et al., 2025). Consistent with this, nutrigenetic studies provide moderate-quality evidence for a dose-dependent reduction in triglyceride levels among male *APOE* ε4 carriers following supplementation with omega-3-rich fish oil (Keathley et al., 2022b). Moreover, because of its critical function in maintaining lipid homeostasis within both the systemic circulation and the central nervous system, *APOE* demonstrates substantial pleiotropy (Zhang et al., 2026). The ε4 allele serves as a primary determinant of cardiometabolic risk while concurrently acting as the most potent genetic risk factor for late-onset Alzheimer's disease, which underscores a deep mechanistic connection between altered lipid metabolism and neurodegeneration (Haapala et al., 2026).

Beyond *APOE*, variation in *PPARA* has been shown to interact with dietary fat intake to influence lipid metabolism. *PPARA* (a master transcriptional regulator of adipogenesis and lipid storage) plays a central role in hepatic lipid and glucose homeostasis, and because polyunsaturated fatty acids act as natural ligands for PPARα, genetic variation at this locus can modify circulating lipid responses, particularly under high-fat or PUFA-rich dietary conditions (Hannon et al., 2018). While *APOE* remains the most prominent locus in lipid nutrigenetics, accumulating evidence suggests that polygenic models incorporating variants in genes such as *ABCA1*, which encodes a cholesterol efflux pump critical for HDL formation, alongside *APOE* explain additional interindividual variability in lipid responses to dietary interventions (Rajendiran et al., 2021).

In the context of glucose metabolism and obesity, SNPs in *TCF7L2*, particularly rs7903146, and *PPARG*, such as rs1801282, are among the most consistently implicated. *TCF7L2* represents the strongest and most reproducibly associated genetic locus for type 2 diabetes risk across diverse populations. It encodes a transcription factor within the Wnt signaling pathway and contains highly conserved functional domains, underscoring its central role in glucose regulation (del Bosque-Plata et al., 2021). Nutrigenetic and observational studies indicate that carriers of *TCF7L2* risk alleles may exhibit differential glycemic and insulin responses to diets varying in fat, fiber, or energy content, particularly in non-diabetic populations, although findings remain heterogeneous and are often not confirmed in randomized dietary interventions (Hosseinpour-Niazi et al., 2022a).

Evidence from population-specific studies further highlights this variability. A recent cross-sectional nutrigenetic study in a Saudi adult cohort reported a near-significant interaction between *TCF7L2* rs7903146 and total energy and saturated fat intake on insulin concentrations, with TT carriers showing greater reductions in insulin levels under lower-energy dietary conditions (Al-odinan et al., 2025). Meanwhile, the *PPARG* rs1801282 variant (commonly known as the Pro12Ala polymorphism) has been associated with differences in adipocyte differentiation, insulin sensitivity, and obesity risk. In a case-control study conducted in a Kazakh population, individuals carrying both the *TCF7L2* rs7903146 TT genotype and the *PPARG* rs1801282 GG genotype exhibited an elevated risk of prediabetes compared with other genotype combinations (Dyussupova et al., 2024). Crucially, the allelic frequencies of these variants vary drastically across ancestries, necessitating caution against over-generalizing findings derived primarily from European cohorts. For instance, the risk allele (T) frequency for *TCF7L2* rs7903146 is approximately 0.29 in European populations, but only 0.03 in Asian populations. Similarly, the *PPARG* rs1801282 variant presents with a minor allele (G) frequency of roughly 0.11 in Europeans, compared to 0.04 in Asians and just 0.01 in African populations (Auton et al., 2015; Gouda et al., 2010; Karczewski et al., 2020).

Importantly, given the broad aim of precision nutrition, interindividual variability extends well beyond macronutrient metabolism to include the processing of dietary bioactives. Recent evidence emphasizes the critical role of genetic variants in determining the bioavailability, biotransformation, and physiological efficacy of plant (poly)phenols. Polymorphisms in genes encoding xenobiotic-metabolizing enzymes and efflux transporters can drastically alter polyphenol pharmacokinetics (Treccani et al., 2025). Consequently, genetic variations dictating how efficiently an individual can metabolize and absorb specific polyphenols serve as a major determinant of their systemic health benefits (Tosi et al., 2025).

Despite these associations, the reproducibility of gene-diet interactions remains a major challenge. Short-term interventions have frequently reported no modification of glycemic outcomes by TCF7L2, whereas longer-term weight-loss interventions occasionally observe genotype-dependent effects (Hosseinpour-Niazi et al., 2022b). To overcome the prevalent reproducibility crisis in gene-diet research, future investigations must transition from observational designs to rigorous, multi-center randomized controlled trials (RCTs) explicitly powered to detect interaction effects. Methodologically, this requires moving beyond subjective food questionnaires by integrating objective nutritional biomarkers and high-resolution metabolomics. Furthermore, given the complex, polygenic nature of metabolic traits, relying on single SNPs often provides limited predictive power. Translating genetic findings into precision nutrition applications will increasingly require the integration of Polygenic Risk Scores (PRS). By aggregating the small effects of thousands of variants across the genome, PRS offer a more comprehensive estimation of an individual's genetic predisposition, ultimately enabling more accurate stratification of responders versus non-responders in tailored dietary interventions (Rami et al., 2026).

### 3.2 Epigenetics in precision nutrition

Epigenetic mechanisms, most notably DNA methylation, post-translational histone modifications, and higher-order chromatin remodeling, provide a dynamic interface by which nutrition and environmental exposures can stably regulate gene expression without altering the underlying DNA sequence. DNA methylation, typically at CpG dinucleotides, can silence or modulate gene transcription. Concurrently, histone modifications, such as acetylation, methylation, and phosphorylation, alter chromatin accessibility. Furthermore, chromatin remodelers reallocate nucleosomes to influence interactions between promoters and enhancers. Together, these epigenetic mechanisms underlie the persistence of diet-induced regulatory states, enabling prior nutritional exposures to influence subsequent regulation of metabolic gene networks (Choi & Friso, 2010; S. X. Li et al., 2017).

Strong evidence for diet-related epigenetic programming arises from studies of maternal nutrition and its effects on the offspring epigenome. During gestation and early postnatal development, periods characterized by heightened epigenetic plasticity, maternal availability of methyl donors and other bioactive nutrients can shape the establishment of epigenetic marks. This process often referred to as nutritional or fetal epigenetic programming. (Najd-Hassan-Bonab et al., 2023). For example, maternal intake of methyl-group donors during the periconception period is associated with differential DNA methylation in infant genes involved in growth, metabolism, and appetite regulation, such as *IGF2*, *LEP* (leptin), *POMC*, and *RXRA* (Jiao et al., 2025). Cohort studies, including the Maternal Nutrition and Offspring's Epigenome (MANOE) study, demonstrate that adequate or supplemental maternal consumption of nutrients such as folate, choline, and B vitamins is linked to altered methylation of imprinted and metabolically relevant genes in offspring, including *IGF2* and *H19*. These findings highlight early pregnancy as a critical window for epigenetic programming with potential implications for long-term metabolic health (Pauwels et al., 2017). Animal models similarly show that a maternal methionine-rich diet induces persistent methylation changes in the offspring's genome, some of which endure into adulthood (Amorín et al., 2023). Maternal overnutrition, particularly high-fat diets, can disrupt epigenetic programming of hypothalamic appetite circuits, increasing offspring susceptibility to obesity through fetal metabolic programming (Harmancıoğlu & Kabaran, 2023). Importantly, epigenetic programming is not exclusively maternal. Recent studies highlight paternal, specifically sperm-mediated, epigenetic effects. A father's preconception diet and metabolic state can alter the DNA methylation and small noncoding RNA profiles of spermatozoa, transmitting metabolic phenotypes such as altered glucose tolerance to the offspring (Laurent et al., 2026).

Beyond early developmental windows, specific dietary interventions in adults have demonstrated the capacity to actively remodel the epigenome. Nutrient-driven epigenetic modulation has been extensively studied for bioactives, essential micronutrients, and microbiota-derived metabolites. For instance, recent intervention studies highlight the potent effects of vitamin D and omega-3 polyunsaturated fatty acids on epigenetic profiles, demonstrating significant alterations in DNA methylation signatures tied to inflammatory and metabolic pathways (Bischoff-Ferrari et al., 2025). Furthermore, specific microbial metabolites such as butyrate, a short-chain fatty acid produced from dietary fiber fermentation, act as endogenous histone deacetylase (HDAC) inhibitors. This inhibition promotes an open chromatin state and facilitates anti-inflammatory gene expression (Rahimah et al., 2026). Similarly, polyphenols such as epigallocatechin gallate, resveratrol, and curcumin have been shown to inhibit DNA methyltransferase activity, alter histone acetylation patterns, and modulate chromatin accessibility in both cellular models and emerging human intervention trials (Borsoi et al., 2023; Lorente-Cebrián et al., 2025; Perlmutter et al., 2024, 2024). Translating these mechanisms to humans, a recent randomized, double-blind, placebo-controlled cross-over trial demonstrated that supplementation with a polyphenol-rich formulation was associated with epigenetic modulation. Specifically, researchers observed hypermethylation at the CpG site cg13108341, a pattern that contrasts with the typical age-related trajectory (Chong et al., 2024).

The consideration of “epigenetic clocks”, validated algorithms that estimate biological age based on specific DNA methylation patterns, offers a powerful tool in precision nutrition (Carvalho et al., 2025). Dietary interventions, particularly those restricting caloric intake or promoting high-quality plant-based diets, have demonstrated the potential to decelerate epigenetic aging relative to chronological age (Janssens et al., 2025). Incorporating these epigenetic clocks into precision nutrition studies provides a quantifiable, longitudinal readout of how specific diets mitigate age-related metabolic decline.

Despite strong mechanistic support, translating epigenetic insights into precision nutrition remains challenging. Evidence from human cohort and intervention studies, including maternal lifestyle interventions during pregnancy, generally shows modest and inconsistent epigenetic changes in offspring, with limited reproducibility across populations. Consistent with these observations, systematic evaluations conclude that the overall certainty of evidence linking maternal dietary patterns to infant epigenetic outcomes remains low. This lack of certainty is largely due to methodological heterogeneity, small sample sizes, and modest effect sizes (Fernando et al., 2023; Panagiotidou et al., 2024). Moving forward, precision nutrition strategies must leverage advanced multi-omics frameworks to standardize adult interventions and validate dynamic epigenetic biomarkers, such as epigenetic clocks, across diverse populations.

### 3.3 MicroRNAs

MicroRNAs are small, approximately 22-nucleotide noncoding RNAs that act as critical post-transcriptional regulators of gene expression. Mechanistically, they primarily bind to complementary sequences on target messenger RNAs (mRNAs) to induce transcript degradation or translational repression (Alaei & Kakumani, 2025). In metabolic contexts, numerous endogenous miRNAs control lipid and glucose metabolism (e.g., miR-33 and miR-103), adipogenesis (e.g., miR-143 and miR-27), and inflammation (e.g., miR-155). The emerging field of nutrimiromics proposes that dietary exposures can influence miRNA expression patterns and thereby shape metabolic phenotypes (DeLucas et al., 2024; Quintanilha et al., 2017).

Among diet-responsive miRNAs, miR-122 is one of the most extensively characterized. Highly enriched in the liver, miR-122 plays a central role in hepatic lipid homeostasis. Its genetic deletion or pharmacological inhibition in animal models leads to reductions in plasma cholesterol, enhanced fatty acid oxidation, and the suppression of lipogenesis (Esau et al., 2006; Wen & Friedman, 2012). In models of diet-induced obesity, miR-122 inhibition has been shown to attenuate hepatic steatosis and improve lipid profiles. This effect likely occurs through the regulation of genes involved in cholesterol and fatty acid synthesis alongside the modulation of metabolic sensor pathways (Hu et al., 2022).

Beyond metabolic regulation, miRNAs contribute significantly to inflammatory signaling. In metabolic and age-related disorders, “inflammamiRs”, including miR-21-5p and miR-146a-5p, are frequently dysregulated. These molecules converge on components of the NF-κB and NLRP3 pathways, sustaining chronic low-grade inflammation in adipose and vascular tissues (Olivieri et al., 2021). For instance, miR-21 is typically induced under inflammatory conditions and can influence Toll-like receptor and NF-κB signaling by targeting transcripts such as *IRAK4*, thereby limiting excessive cytokine production in macrophages (Chu et al., 2019). Similarly, miR-146a-5p interacts with inflammatory mediators such as IL-6, highlighting their coordinated roles in metaflammation associated with aging and metabolic disease (Faraldi et al., 2025; Mortsch et al., 2026).

Given their capacity to integrate upstream dietary cues with downstream regulatory effects, miRNAs are emerging as potential biomarkers of nutritional response. Circulating levels of miR-122 have been associated with features of metabolic syndrome. In dietary weight-loss interventions, its reduction correlates with improvements in metabolic parameters (Hess et al., 2020). A recent scoping review highlighted the scarcity of robust human trials in this area by identifying only 25 eligible studies out of over 6000 screened. Despite this limited evidence base, the review noted some consistency for miR-21-5p as a responsive marker to dietary interventions in metabolic and inflammatory settings. However, the authors concluded that substantially more rigorous research is required (Chodur & Steinberg, 2024). Furthermore, the broad expression of miR-21 across various tissues limits its specificity as a dedicated nutrition-related biomarker (Jenike & Halushka, 2021).

The complexity of these regulatory networks is further exemplified by the modulation of the miR-21 axis by dietary polyphenols. Resveratrol, for example, downregulates miR-21 expression, thereby relieving the translational repression of its direct target, phosphatase and tensin homolog (PTEN). The resulting accumulation of PTEN antagonizes the PI3K/AKT signaling cascade, ultimately reducing oxidative stress and attenuating NF-κB-driven inflammation (Ghafouri-Fard et al., 2022; Yan et al., 2018). This demonstrates how a single functional food component can act through a specific miRNA node to promote a comprehensive metabolic shift.

Crucially, the scope of nutrimiromics has recently expanded beyond the modulation of endogenous host miRNAs to include the direct physiological actions of food-derived miRNAs. Cutting-edge research reveals that exogenous miRNAs derived directly from dietary sources, often encapsulated within plant- or animal-derived extracellular vesicles, can survive gastrointestinal digestion. These miRNAs can then enter the host's systemic circulation, and actively regulate host gene expression (Leroux et al., 2026). This cross-kingdom epigenetic regulation positions food-derived miRNAs not merely as metabolic byproducts, but as biologically active signaling molecules capable of transferring regulatory information directly from diet to host. Advancing this field will require integrative study designs that combine miRNA profiling with other omics layers, such as transcriptomics and metabolomics, to strengthen causal inference and support biomarker validation.

## 4 Microbiome–gene–diet interactions

**Fig. 3:**
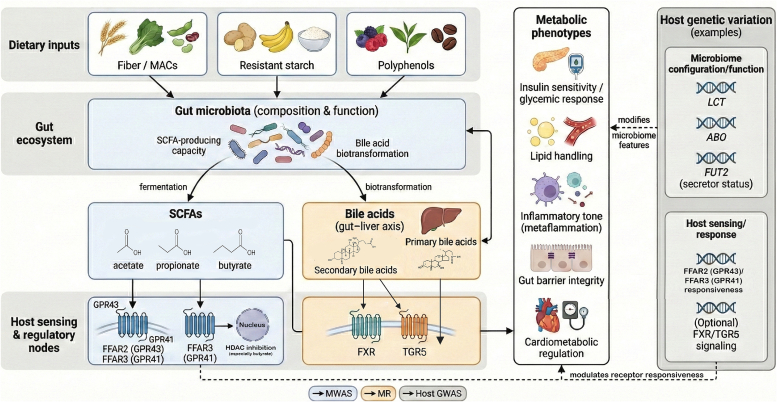
Dietary substrates (e.g., fiber/MACs, resistant starch, polyphenols) shape gut microbiota function and metabolite production, including SCFAs (acetate, propionate, butyrate) and microbiota-modified bile acids in the gut–liver axis. SCFAs signal via *FFAR2* (GPR43) and *FFAR3* (GPR41) and via butyrate-mediated HDAC inhibition, while bile acids act through FXR and TGR5, collectively influencing glycemic control, lipid handling, inflammation, barrier integrity, and cardiometabolic regulation. Host genetic variation (e.g., *LCT*/*ABO*/*FUT2*) can modulate microbiome features and receptor responsiveness. Evidence streams summarized include microbiome-wide association studies (MWAS), Mendelian randomization (MR) for causal inference, and host genome-wide association studies (GWAS).

The gut microbiota is a central determinant of how diet and host genetics jointly shape metabolic outcomes (Fig. 3). Through the fermentation of otherwise indigestible substrates, including dietary fiber, resistant starch, and other microbiota-accessible carbohydrates (MACs), intestinal microbes produce bioactive metabolites such as SCFAs. Additionally, Additionally, microbes modify endogenous host compounds to generate secondary bile acids and other small molecules that act as signaling intermediates for the host (Ayakdaş & Ağagündüz, 2023; Carmody et al., 2024; Chen et al., 2024; Read & Holmes, 2017). These products influence insulin sensitivity, lipid handling, and inflammatory tone via pathways that include SCFA-sensing G protein-coupled receptors (GPCRs), such as GPR41/FFAR3 and GPR43/FFAR2. They also exert epigenetic regulation through HDAC inhibition (Chen et al., 2024; Lee et al., 2024). A major complementary route is the gut-liver axis. Here, diet reshapes the size and composition of the bile acid pool, while microbial biotransformation shifts the balance of primary versus secondary bile acids. These changes subsequently modulate receptor signaling through FXR and TGR5, exerting downstream effects on barrier integrity, immune tone, and cardiometabolic regulation (Fleishman & Kumar, 2024; Y. Li et al., 2024; Song et al., 2025). These mechanisms explain why dietary perturbations may modify, amplify, or buffer the phenotypic expression of genetic predisposition across individuals and populations (Carmody et al., 2024; Qin et al., 2022).

Metagenomic and microbiome-wide association studies increasingly support microbiome-host genetic interactions in metabolic phenotypes, although directionality is often difficult to infer from observational data alone. Genetic causal inference helps clarify this relationship. Bidirectional Mendelian randomization using host genetic instruments linked to microbiome-related SCFAs indicates that genetically driven higher butyrate production is associated with improved insulin response during an oral glucose tolerance test (OGTT). Conversely, abnormalities in propionate production or absorption are causally linked to increased type 2 diabetes risk (Sanna et al., 2019). Complementing these findings, a large host GWAS in the Dutch Microbiome Project (7738 participants) shows that host genetic effects on microbial taxa and pathways are detectable but generally modest. The strongest replicated signals near the *LCT* and *ABO*. These signals are modulated by lactose intake and partly explained by secretor status (*FUT2*). This underscores the diet-dependent nature of these interactions and the need for larger cohorts to map additional loci (Lopera-Maya et al., 2022).

At a broader level, combined host genetic and dietary factors explain a meaningful fraction of interindividual variation in microbiome diversity and metabolite profiles. For instance, host genetic variants can dictate the baseline colonization of key microbial taxa. This baseline subsequently shapes how heavily dietary interventions influence the abundance of specific short-chain fatty acid (SCFA)-producing bacteria, such as *Bifidobacterium* and *Faecalibacterium* (Huda et al., 2022). Global metagenomic profiling of SCFA-production potential suggests that lifestyle-associated differences in ecological redundancy among SCFA-encoding microbes exist across populations. This implies that some microbiomes may be more resilient to disruption in SCFA production than others (Jacobson et al., 2021). Such functional resilience could plausibly influence how dietary exposures interact with host genetic susceptibility to shape metabolic phenotypes.

Translating this ecological redundancy into metabolic benefits depends on genetic and epigenetic variations in SCFA-sensing GPCRs. For instance, UK Biobank analyses link human variants causing lower *FFAR2* and *FFAR3* expression to cardiometabolic risks. Simultaneously, altered *FFAR3* promoter methylation correlates with differences in BMI and type 2 diabetes profiles. Despite this strong observational and mechanistic evidence, these specific host genetic signatures have not yet been utilized to stratify large-scale precision nutrition clinical trials. Consequently, clinical validation of *FFAR2* and *FFAR3* markers to guide personalized prebiotic interventions remains an unfulfilled translational gap (Muralitharan et al., 2025; Remely et al., 2014).

Consistent with this, an integrative precision nutrition paradigm proposes that diet responses depend on host genotype, microbiome structure, and metabolic capacity. Therefore, diet-microbiome-host genetics should be analyzed as an interconnected system rather than independent components (Carmody et al., 2024; Livingstone et al., 2022). In this model, dietary patterns (e.g., high-fiber or polyphenol-rich diets) can shift microbiome-derived signaling, including SCFAs and bile-acid derivatives. Concurrently, host variation in sensing and downstream pathways may condition the metabolic impact of comparable microbial exposures (Carmody et al., 2024; Zhernakova et al., 2024). For example, if genetic variation reduces sensitivity in SCFA-sensing GPCR pathways (*FFAR2*/*FFAR3*), similar increases in microbiome-derived SCFAs may yield smaller metabolic benefits than in individuals with more responsive alleles. This illustrates why multi-layer integration can improve prediction (Lee et al., 2024; Qin et al., 2022; Tavares et al., 2024). Accordingly, next-generation frameworks increasingly emphasize integrating host genomic data with metagenomic and metabolomic microbiome profiling alongside quantified dietary assessment to strengthen mechanistic inference and personalization (Carmody et al., 2024; Livingstone, Ramos-López, et al., 2022; Zhernakova et al., 2024).

Despite these advances, major limitations remain. Many microbiome association studies are cross-sectional and cannot disentangle causal pathways among diet, microbiome, genotype, and metabolic phenotype. Furthermore, confounding variables and complex microbe-microbe interaction networks can generate statistical artifacts and spurious associations (Menon et al., 2017). Progress will require intervention trials that stratify participants by genotype and baseline microbiome. These trials must also longitudinally quantify metagenomic and metabolomic shifts alongside metabolic endpoints.

## 5 Clinical and public health evidence

Large-scale cohort studies offer foundational epidemiologic evidence linking diet, genetic variation, and health outcomes in populations. The UK Biobank is a large prospective cohort of approximately 500,000 adults recruited between 2006 and 2010. This resource integrates dietary questionnaires, repeated 24-h recalls in participant subsets, biomarker and genomic data, and longitudinal health outcomes (Fry et al., 2017). Across analyses and reviews, healthier dietary patterns are generally associated with modestly lower risks of type 2 diabetes, cardiovascular disease, and some cancers. However, relying on one-time dietary assessments in many studies may introduce measurement error and attenuate effect estimates (Navratilova et al., 2024; Shang et al., 2023). The UK Biobank also supports research on gene and diet interactions alongside precision nutrition modeling by linking genotype with detailed lifestyle and clinical outcomes (Westerman et al., 2021). Complementing cohort evidence, postprandial phenotyping initiatives such as the PREDICT study quantify individual metabolic responses under controlled feeding conditions. These studies relate glycemic, lipemic, and inflammatory variability to genomic, microbiome, and metabolomic profiles, offering a pragmatic framework for individualized response prediction (Bermingham et al., 2023; Berry et al., 2020). Together, these approaches connect real-world association signals with mechanistically informative response phenotypes.

In intervention research, trials incorporating genotype or epigenetic stratification remain in early stage. Retrospective analyses have tested whether variants in genes such as *APOE*, *FTO*, and *PPARG* modify responses to dietary fat composition, Mediterranean-style diets, or macronutrient restriction. However, limited statistical power and the frequent lack of prospective genotype-based stratification constrain causal inference (De Toro-Martín et al., 2017; Livingstone, Ramos-Lopez, et al., 2022; Mansour et al., 2024). A small number of pilot trials has also evaluated epigenetic endpoints, such as DNA methylation changes, after supplementation with methyl-donor nutrients or polyphenol-rich foods. Yet short study duration and small sample sizes limit generalizability (Mehta et al., 2024).

From a clinical and public health perspective, precision nutrition could improve dietary prescribing and risk stratification. It may also reduce trial-and-error approach in counseling across chronic conditions including obesity, type 2 diabetes, cardiovascular disease, nonalcoholic fatty liver disease, and cancer (Mehta et al., 2024; V. K. Singh et al., 2024; Voruganti, 2023). However, translation at scale is constrained by modest single-variant effect sizes, ancestry- and context-specific heterogeneity, and the cost and scalability of omics profiling. Additional barriers include ethical and privacy considerations, as well as the need for validation in diverse populations. Successful translation also requires implementation requirements for health-system integration, behavioral support, and appropriate oversight (Livingstone, Ramos-López, et al., 2022; Mehta et al., 2024; Voruganti, 2023). The next step is to advance from observational modeling and feasibility studies to well-powered, genotype- or epigenotype-informed randomized trials across diverse ancestries. Ultimately, the goal is to translate omics-derived insights into actionable, cost-effective dietary interventions.

## 6 Data integration, AI, and translation pathways in precision nutrition

**Fig. 4:**
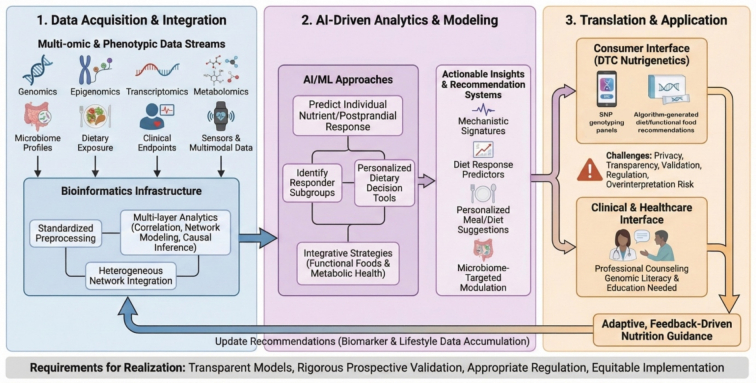
Data integration and AI-enabled translation in precision nutrition. Multi-omic, dietary, clinical, and sensor-derived data are integrated using bioinformatics pipelines and multi-layer analytics to derive predictors of diet response. AI/ML models support response prediction, responder stratification, and personalized dietary decision tools, with translation through clinical and direct-to-consumer pathways and iterative updating as new data accrue.

Precision nutrition is increasingly enabled by mature bioinformatics infrastructures that integrate multi-omics and phenotypic data streams, including genomics, epigenomics, transcriptomics, metabolomics, microbiome profiles, dietary exposure, and clinical endpoints (Nourazarain & Vaziri, 2025; Zhu et al., 2025). These pipelines typically rely on standardized preprocessing step, such as the normalization of omics data, batch correction, and feature scaling. Researchers utilize these processes alongside multi-layer analytics, including hierarchical models and multi-omics integration frameworks to identify mechanistic signatures and predictors of diet response (Zhu et al., 2025; Zitnik et al., 2023). Such analytics routinely employ correlation and network modeling, such as Bayesian networks to map complex variable dependencies. They also utilize heterogeneous network integration to combine disparate biological layers into a unified graph. Furthermore, investigators apply causal inference techniques, such as Mendelian randomization, to distinguish true causal dietary drivers from confounding factors. In parallel, artificial intelligence and machine learning (AI/ML) approaches are being applied to predict individual nutrient responses, identify responder subgroups, and support personalized dietary decision tools, although prospective clinical validation remains limited (Wu et al., 2025). Fig. 4 summarizes the end-to-end data-to-decision workflow and key translation pathways in precision nutrition.

A major barrier to the clinical translation of these predictive models is their inherent “black box” nature. Complex algorithms, such as deep neural networks or ensemble methods, often obscure the biological rationale behind their outputs (Alum et al., 2026; Sharma et al., 2025). For healthcare professionals to trust and safely implement AI-generated guidance, there is a critical need for Explainable AI (XAI) (Liu et al., 2025). XAI frameworks aim to make model predictions transparent by explicitly identifying which specific multi-omic features drove a given dietary recommendation. These features might include a particular genetic variant (Ren et al., 2026), a microbiome-derived metabolite (Rozera et al., 2025), or a baseline glycemic marker (Mukherjee & De, 2026). Integrating XAI into precision nutrition pipelines is therefore essential for bridging the gap between computational prediction and clinical reasoning, ensuring that AI tools augment rather than replace clinical judgment.

Proof-of-concept studies indicate that multi-input models integrating diet, microbiome, and host features can forecast interindividual postprandial glycemic and lipemic responses. This capability supports the feasibility of actionable personalization through improved phenotype prediction and more refined patient stratification (Berry et al., 2020). Furthermore, emerging systems incorporating wearable sensors, such as continuous glucose monitors and smartwatches. These devices work alongside multimodal data like digital food logs and clinical biometrics, aim to improve dietary assessment and tracking accuracy (Arefeen et al., 2025). AI-enabled recommendation platforms have also been piloted to guide microbiome-targeted dietary modulation, though most evidence remains early-stage (Rouskas et al., 2025; Theodore Armand et al., 2024).

The rapid expansion of AI applications in precision nutrition has accelerated the development of concrete, real-world decision-support applications. For example, digital twins for metabolic simulation allow researchers and clinicians to test dietary interventions in silico before application. Simultaneously, automated systems generate personalized meal suggestions from individual profile data and constraints (Kalpakoglou et al., 2025; Tsolakidis et al., 2024; Wu et al., 2025). Within the context of functional foods and metabolic health, applying these integrative strategies helps map specific bioactive exposures to affected molecular axes under defined genetic or epigenetic conditions. This mapping enables more mechanistically grounded personalization (Nourazarain & Vaziri, 2025; Zitnik et al., 2023).

At the consumer interface, direct-to-consumer (DTC) nutrigenetic services have expanded rapidly. These commercial platforms analyze specific individual genetic variants to provide personalized dietary advice, have expanded rapidly, typically using SNP panels and composite scores linked to proprietary algorithms to generate diet or functional-food recommendations (Floris et al., 2020). Crucially, however, because the gut microbiome profoundly conditions dietary outcomes independent of host genetics. Consequently, purely nutrigenetic-based services cannot comprehensively capture the full biological impact of diet on the host. Furthermore, reviews highlight persistent concerns regarding privacy, limited transparency of models and validation datasets. Companies frequently utilize proprietary “black box” algorithms that obscure how genetic markers are weighted, and they rarely disclose their validation datasets. This lack of transparency, combined with inconsistent regulatory oversight, creates the risk that probabilistic results are overinterpreted without appropriate counseling. This overinterpretation could potentially lead to consumer harm (Ceriani et al., 2023; Duarte et al., 2024; Jiang et al., 2023). These issues underscore the need to strengthen genomic literacy and continuing education among healthcare professionals, who increasingly encounter DTC results in practice (Martins et al., 2022).

The convergence of integrative bioinformatics, AI/ML, and scalable digital platforms could shift the field from static associations to adaptive, feedback-driven nutrition guidance. Such guidance would update recommendations as biomarker and lifestyle data accumulate (Agrawal et al., 2025; Rouskas et al., 2025). Realizing this potential will require transparent and interpretable models, rigorous prospective validation, appropriate regulation, and equitable implementation alongside technical innovation.

## 7 Challenges and limitations

A persistent limitation in nutritional epigenetic research is reproducibility. Many reported gene-diet interactions and diet-associated epigenetic signals have not been replicated across independent cohorts. This lack of replication reflects modest effect sizes, multiple testing, and dependencies specific to distinct populations or contexts. Systematic evaluations note that gene-nutrient interactions for lipid traits, including those involving omega-3 exposures, frequently show inconsistent replication and limited clinical magnitude (Keathley et al., 2022a; Wuni & Vimaleswaran, 2024). More broadly, heterogeneity in dietary assessment, analytic flexibility, and selective reporting can inflate false positives (Sorkin et al., 2016). These issues underscore the need for adequately powered, pre-registered studies with pre-specified hypotheses. Furthermore, researchers must implement standardized protocols, transparent reporting, and data sharing to enable robust external validation.

Study design constraints further limit statistical inference. Many nutrigenetic and epigenetic studies have small sample sizes, reducing the statistical power to detect modest interaction effects or subtle epigenetic changes (V. Singh, 2023; Wuni & Vimaleswaran, 2024). Heterogeneity in ancestry, baseline diet, lifestyle, and comorbidities can introduce confounding variables and limit generalizability. Additionally, unmeasured exposures, medication use, and microbiome differences may distort inferred genotype-diet or epigenotype-diet relationships. Furthermore, many trials are short-term and rely on surrogate biomarkers rather than hard clinical endpoints, which constrains translational relevance (Surendran & Vimaleswaran, 2021; Wuni & Vimaleswaran, 2024; Zoh et al., 2023).

Ethical, legal, and privacy concerns add another layer of complexity. The use of genomic and epigenomic data raises issues of confidentiality, informed consent (particularly regarding incidental findings), data sharing, and potential genetic discrimination (Ceriani et al., 2023; Francis, 2014; Reilly & Debusk, 2008). In nutrigenetic applications, critics also warn against overstating the certainty of genotype-based dietary advice. This particularly true when provider training and counseling are limited, which increases the risk of misinterpretation and psychological harm (Beckett et al., 2017; Ceriani et al., 2023). Regulatory protections for genetic privacy and the oversight of direct-to-consumer genotype-based nutrition services remain incomplete or inconsistent across jurisdictions (Bonomi et al., 2020; Callier et al., 2016; Horne et al., 2020; Nicol et al., 2024).

Finally, the bench-to-bedside gap remains substantial. Many mechanistic findings from cellular and animal models have not been validated in humans, especially across diverse ancestries. Clinical adoption is further constrained by the cost and complexity of multi-omics profiling and the limited interoperability of analytic platforms. Significant gaps also exist in clinician and dietitian training regarding genomic and epigenomic interpretation (Murgia & Adamski, 2017; V. Singh, 2023). Bridging this gap will require large, multicenter trials stratified by genotype and epigenotype. It will also necessitate validated and interpretable predictive models, scalable bioinformatic infrastructure, and coordinated efforts in education, reimbursement, and stakeholder engagement.

## 8 Future prospects

Future progress in precision nutrition will likely be driven by the comprehensive integration of multi-omics layers, such as genomics, epigenomics, transcriptomics, proteomics, metabolomics, and microbiome profiling. These molecular layers must be combined with longitudinal dietary and lifestyle data. Such integrated designs can help resolve causal pathways linking dietary exposures to molecular intermediates and, ultimately, clinical phenotypes. This integration clarifies the biological basis of interindividual variability in nutrient response (Mani et al., 2025). Systems-biology studies are increasingly leveraging this paradigm. Researchers are applying machine learning and network-based modeling to derive actionable biomarkers and dietary signatures from multi-omics inputs (Ramos-Lopez et al., 2022). In metabolic health, these approaches could define robust metabotypes or response phenotypes. These profiles would enable individualized, food- and nutrient-specific dietary recommendations that extend beyond broad dietary categories.

Simultaneously, digital nutrition tools and wearable biosensors are poised to connect relatively static molecular profiling with real-time monitoring and feedback. Emerging platforms enable continuous or near-continuous measurement of physiological and biochemical signals, such as glucose, interstitial fluid, and sweat biomarkers. They also capture complementary metrics including heart rate variability, with improving sensitivity and user compatibility (Sempionatto et al., 2021; Yuan et al., 2023). Advances in flexible microfluidic and graphene-based sensor technologies further expand the feasibility of continuous monitoring in everyday settings (Abdelfattah et al., 2025). Machine learning can facilitate multimodal data fusion and inference within these sensor networks. For example, systems integrating multiple sensor streams and diet tracking (Arefeen et al., 2025) could deliver dynamic, context-aware nutritional recommendations. Such guidance would adapt in real time as individuals eat, move, or respond to metabolic shifts (Tahtouh et al., 2025).

A further emerging frontier is the potential role of miRNA-based modulation within precision nutrition. Because miRNAs function as network-level regulators of gene expression, shaping their expression through diet could be highly impactful. Directly targeting specific miRNAs, such as miR-122 and miR-21, could potentially influence metabolic and inflammatory pathways in a selective manner (Diener et al., 2022; Niazi & Magoola, 2024). Notably, antisense oligonucleotides and miRNA mimics/antagomirs are already being evaluated in other therapeutic contexts, including liver disease and cancer (di Martino et al., 2025). Translating such strategies to metabolic nutrition would require careful genotype-informed stratification, effective delivery approaches, and rigorous safety and efficacy validation in appropriate metabolic models. Although still speculative, miRNA-directed approaches could ultimately complement dietary and functional food interventions. This combination would be particularly valuable for individuals in whom diet alone produces limited benefit.

## 9 Conclusion

Precision nutrition is a developing framework for personalizing dietary interventions using interindividual molecular variability, including DNA sequence variation, *epi*genetic regulation, and miRNA-mediated control. By accounting for heterogeneous responses to nutrients and functional foods, it provides a mechanistic basis for individualized strategies to support metabolic health. Integrating nutrigenetics, nutri*epi*genomics, and nutrimiromics helps explain divergent responses to the same dietary exposure. This integration also highlights candidate biomarkers and pathways for stratified intervention design. The evidence base remains mixed. While large cohorts and controlled studies demonstrate feasibility, reported effects are often modest, and reproducibility varies across populations. Many gene-diet interactions and epigenetic or miRNA signatures remain exploratory. This uncertainty necessitates adequately powered studies, standardized measurement of diet and outcomes, and transparent analytics. Additionally, findings must be replicated across diverse ancestries and real-world contexts.

Clinical translation will require prospective trials informed by genotypes and epigenotypes clinically meaningful endpoints. These efforts must operate alongside robust data governance, privacy protections, and the responsible communication of probabilistic risk. Training for clinicians and dietitians, as well as the oversight of consumer-facing genetic services will be essential to prevent misinterpretation and inequitable implementation. The most credible path to impact is the convergence of multi-omics with digital health. Coupling omics profiling with longitudinal dietary assessment, wearable biosensors, and AI-enabled decision support could enable adaptive recommendations. These guidelines would continuously update with changing physiology and exposures. With rigorous validation, interpretability, and appropriate ethical and regulatory frameworks, precision nutrition could mature from a promising concept into a scalable approach for improving metabolic health in clinical and public health settings.

## Declaration of the use of generative artificial intelligence

During the preparation of this manuscript, the authors used ChatGPT (version 5.2) to assist with improvements in spelling, grammar, clarity, and readability of the text. Following the use of this tool, the authors critically reviewed and edited all content as necessary and take full responsibility for the integrity, originality, and accuracy of the work presented.

## CRediT authorship contribution statement

**Souvia Rahimah:** Writing – review & editing, Writing – original draft, Visualization, Project administration, Methodology, Investigation, Funding acquisition, Data curation, Conceptualization. **Trina Ekawati Tallei:** Writing – review & editing, Writing – original draft, Visualization, Supervision, Project administration, Methodology, Investigation, Funding acquisition, Conceptualization. **Maghfirah Savitri:** Writing – review & editing, Writing – original draft, Visualization, Methodology, Investigation, Formal analysis, Data curation. **Chika Yamada:** Writing – review & editing, Investigation, Formal analysis. **Hyo Jung Kim:** Writing – review & editing, Resources. **Min Choi:** Writing – review & editing, Resources. **Moon Nyeo Park:** Writing – review & editing, Resources. **Youdiil Ophinni:** Writing – review & editing, Supervision, Formal analysis. **Bonglee Kim:** Writing – review & editing, Supervision.

## Funding statement

The publication charge was funded by Universitas Padjadjaran through the Indonesian Endowment Fund for Education (LPDP) on behalf of Indonesian Ministry of Higher Education, Science and Technology and manage under the EQUITY Program (Contract No. 4303/B3/DT.03.08/2025 and 3985/UN6.3.1/PT.00/2025).

## Declaration of competing interest

The authors declare that they have no known competing financial interests or personal relationships that could have appeared to influence the work reported in this paper.

## Data Availability

No new data were generated or analyzed in this study. Data sharing is not applicable to this article.
